# Toward Patient-Centered Telerehabilitation Design: Understanding Chronic Pain Patients’ Preferences for Web-Based Exercise Telerehabilitation Using a Discrete Choice Experiment

**DOI:** 10.2196/jmir.5951

**Published:** 2017-01-20

**Authors:** Karlijn Cranen, Catharina GM Groothuis-Oudshoorn, Miriam MR Vollenbroek-Hutten, Maarten J IJzerman

**Affiliations:** ^1^ Roessingh Research and Development Telemedicine group Enschede Netherlands; ^2^ Department of Health Technology and Services Research University of Twente Enschede Netherlands; ^3^ Ziekenhuisgroep Twente Almelo Netherlands; ^4^ Faculty of Electrical Engineering, Mathematics and Computer Science, Telemedicine group University of Twente Enschede Netherlands

**Keywords:** patient preference, patient acceptance of health care, telerehabilitation, choice behavior, decision making, decision support techniques, patient compliance, chronic disease, exercise therapy, chronic pain

## Abstract

**Background:**

Patient-centered design that addresses patients’ preferences and needs is considered an important aim for improving health care systems. At present, within the field of pain rehabilitation, patients’ preferences regarding telerehabilitation remain scarcely explored and little is known about the optimal combination between human and electronic contact from the patients’ perspective. In addition, limited evidence is available about the best way to explore patients’ preferences. Therefore, the assessment of patients’ preferences regarding telemedicine is an important step toward the design of effective patient-centered care.

**Objective:**

To identify which telerehabilitation treatment options patients with chronic pain are most likely to accept as alternatives to conventional rehabilitation and assess which treatment attributes are most important to them.

**Methods:**

A discrete choice experiment with 15 choice tasks, combining 6 telerehabilitation treatment characteristics, was designed. Each choice task consisted of 2 hypothetical treatment scenarios and 1 opt-out scenario. Relative attribute importance was estimated using a bivariate probit regression analysis. One hundred and thirty surveys were received, of which 104 were usable questionnaires; thus, resulting in a total of 1547 observations.

**Results:**

Physician communication mode, the use of feedback and monitoring technology (FMT), and exercise location were key drivers of patients’ treatment preferences (*P*<.001). Patients were willing to accept less frequent physician consultation offered mainly through video communication, provided that they were offered FMT and some face-to-face consultation and could exercise outside their home environment at flexible exercise hours. Home-based telerehabilitation scenarios with minimal physician supervision were the least preferred. A reduction in health care premiums would make these telerehabilitation scenarios as attractive as conventional clinic-based rehabilitation.

**Conclusions:**

“Intermediate” telerehabilitation treatments offering FMT, some face-to-face consulting, and a gym-based exercise location should be pursued as promising alternatives to conventional chronic pain rehabilitation. Further research is necessary to explore whether strategies other than health care premium reductions could also increase the value of home telerehabilitation treatment.

## Introduction

### Chronic Pain and Treatment

Chronic pain is considered a major public health problem. Breivik et al [1] explored the prevalence of chronic pain in 15 European countries and Israel and found that 19% (N=8.815) of their study sample suffered from chronic pain varying from moderate to severe intensity. Due to an aging society, it is expected that the prevalence of chronic pain may rise even higher, as chronic pain prevalence is greater in older adults [2,3]. Chronic pain often interferes with family and home responsibilities, recreational activities [1], and sleep [4], and it is linked with an increased risk of depression [5]. In addition to the physical and emotional burden chronic pain brings, it accounts for considerable direct health care costs, including costs related to tests, medication, and treatment, as well as indirect costs such as lost income and reduced work productivity [6]. In European countries, pain is estimated to cost economies between 3% and 10% of gross domestic products [4], resulting in an estimate of at least €140 billion [7].

Physical training has been proven to decrease pain and improve function [8-10] and therefore plays an important role in current (multidisciplinary) pain rehabilitation programs. The majority of these programs are clinic-based and supervised [11]. Although conventional rehabilitation programs are effective, poor adherence and high relapse have been shown to compromise the effectiveness of these programs [11-14] and as such lead to increased costs [15].

### Patient-Centered Design

An important factor in facilitating treatment adherence is the design of patient-centered treatment programs [16-18]. The Institute of Medicine defines patient-centered care as “providing care that is respectful of, and responsive to, individual patient preferences, needs and values, and ensuring that patient values guide all clinical decisions” [19]. The concept of patient-centered care has received increased attention in recent years and is considered an important aim for health care system improvement [19,20].

Clinical guidelines for the management of chronic pain follow up on this patient-centered approach and recommend that patient preferences should be considered and that treatment programs should be individualized [21]. The underlying assumption is that by designing programs that address patients’ preferences and beliefs, treatment adherence will improve [22]. In addition, there is evidence that patient preferences affect treatment outcome. A systematic review found an increase in the effectiveness of the treatment among participants in musculoskeletal medicine trials, who were randomized to their preferred treatment compared with those who were indifferent to the treatment allocation [23]. In addition, patients’ preferences should be respected on the basis of moral grounds alone regardless of their relationship to the health outcomes [24].

The assessment of chronic pain patients’ preferences is, therefore, a necessary first step toward the design of patient-centered pain rehabilitation programs that help better meet patients’ needs. The gap between what patients prefer and what is offered can be identified, and treatment may be optimized [22].

One method to estimate patients’ preferences is the use of a discrete choice experiment (DCE). A DCE is a preference elicitation methodology that is being increasingly used in health care research [25,26]. Respondents are offered a series of choices between 2 or more treatment alternatives, described by a combination of treatment attributes, and choose their preferred treatment. Analysis of these choices allows for the estimation of the relative importance of treatment attributes. A DCE can assist in prioritizing health care resource allocation, as it provides a better understanding of the factors that are most important to patients and can be used to inform patient-centered telerehabilitation design. In addition, the use of DCEs is especially valuable in the context of innovative treatments, for example, chronic pain telerehabilitation treatment, as it allows for the estimation of patients’ preferences for multiple treatment scenarios that do not yet exist.

### Telerehabilitation

In recent years, the use of telerehabilitation, providing remote delivery of rehabilitative services through Internet and communication technology, has been steadily increasing [27]. Systematic reviews have demonstrated that telerehabilitation has small but significant effects on pain experience and reduction in functional disability [28-30]. A review by Kairy et al [27] concluded that telerehabilitation can lead to clinical outcomes that are similar to those of traditional rehabilitation programs. Telerehabilitation is considered a promising alternative strategy next to conventional clinic-based rehabilitation programs, as it can facilitate access and adherence to health interventions [31]. Since pain rehabilitation involves changes in often long-lasting personal behavior and lifestyle, it is important that patients are able to use the acquired skills outside of the rehabilitation clinic. However, as most rehabilitation programs are supervised and provided in clinics, they may not be conducive to fostering maintenance or compliance in patients’ natural environments [11]. Telerehabilitation, offering care in the patients’ environment, can be a better fit with the patient’s lifestyle, and by doing so, translation of the acquired skills into the patients’ environment will become easier [16,32]. Furthermore, telerehabilitation has the potential to foster patient self-management [33]. For example, performance can be monitored and feedback can be provided on progress without the real-time involvement of a therapist, which perhaps will empower patients to take an active role in their own rehabilitation [34]. Self-management is especially encouraged in patients with a long-term condition such as chronic pain and has been shown to improve patient outcomes [35]. International clinical practice guidelines endorse the promotion of self-management behavior, including physical activity, for chronic pain patients as an important component of care [21,36]. In a systematic review, Liddle et al [37] found that educating chronic pain patients about appropriate exercise and function activity to promote active self-management is effective.

At present, within the field of pain rehabilitation, patients’ preferences of telerehabilitation remain scarcely explored and little is known about the optimal combination between human and electronic contact from the patients’ perspective. In addition, limited evidence is available about the best way to explore patients’ preferences. To our knowledge, this is the first study in the field of telemedicine that uses a DCE to explore what patients want as well as explore their priorities. As telerehabilitation represents a fundamental change from conventional treatment programs, it is vital to understand patients’ preferences, and DCEs may prove to be invaluable, as the market potential of different prospective telerehabilitation services can be simulated.

Therefore, this study aims to identify chronic pain patients’ preferences for telerehabilitation services using a DCE. The primary objective is to determine what treatment attributes are most important to chronic pain patients and identify which telerehabilitation scenario chronic pain patients are most likely to accept as an alternative to conventional rehabilitation. Conventional rehabilitation was described as physical activity through supervised group exercise at the clinic. The telerehabilitation scenarios that were explored varied at different levels, allowing exploration of the potential benefit of telerehabilitation. Jansen-Kosterink [38] states that the potential value of telemedicine services depends on the technology used, the clinical purpose it serves, and how the telemedicine service is implemented in daily clinic practice (service configuration). To that end, the scenarios explored different types of technology used for different clinical purposes (eg, monitoring or coaching) and also explored different methods of service configuration (eg, clinic-based care or home-based treatment). The scenarios represented a continuum of health care services ranging from clinic- based rehabilitation to home-based telerehabilitation with a focus on patient self-management. Furthermore, a willingness to accept (WTA) was estimated to explore whether patients were willing to trade health care premium reduction for more resource-efficient telerehabilitation treatments. To our knowledge, this is the first study in the field of telerehabilitation to assess patients’ preferences with a DCE.

## Methods

### Study Design

Implemented as part of a larger survey that explored patients’ attitudes toward telerehabilitation, patients’ preferences for hypothetical telemedicine treatments were elicited using a self-administered discrete choice survey. The discrete choice experiment followed the International Society for Pharmacoeconomics and Outcomes Research (ISPOR) checklist [39] on patient-preference methods. The following steps were taken: (1) identification of the key treatment attributes and assignment of levels to the attributes; (2) design of the experiment and determination of hypothetical treatment scenarios using various combinations of attributes and levels; (3) choosing an elicitation format and obtaining choice data in patients; and (4) analysis of the choice data. These steps are described in the following section.

### Identification of Key Attributes of Telemedicine Treatment and Assignment of Levels

Qualitative interviews with 10 chronic pain patients (6 females, mean age 41.0 years, with pain complaints lasting longer than 6 months) and an expert focus group with 6 professionals (4 rehabilitation therapists, 1 nurse practitioner, and 1 rehabilitation doctor) were used to select the following attributes (Table 1) for inclusion in the survey: (1) treatment mode and location, (2) physician contact mode, (3) physician contact frequency, (4) feedback and monitoring technology, (5) program flexibility, and (6) health care premium reduction. The health care premium reduction attribute was used to estimate a “willingness to accept” value. This value represented a reduction in health care premiums and was used to explore whether patients were willing to trade more expensive conventional rehabilitation services for premium reductions.

Using the 6 attributes, a pilot questionnaire was developed and tested on 15 patients (11 females, mean age 42.5 years, with pain complaints lasting longer than 6 months) attending treatment in the rehabilitation clinic. In the pilot, data were collected on the time taken to complete the questionnaire and the patients’ understanding of the questionnaire. Only minor adaptations were made after the pilot tests, in particular regarding the wording of the attributes.

**Table 1:** Treatment attributes and levels used to construct the rehabilitation scenarios.

Attribute	Levels
Treatment mode and location	You exercise in a group at the gym
	You exercise individually at the gym
	You exercise individually at home
	You exercise in a virtual group at home
Physician contact mode	All physician contact takes place at the clinic face-to-face
	One quarter of your physician contact through Web camera
	Three-quarters of your physician contact through Web camera
	All your physician contact takes place through Web camera
Physician contact frequency	Every exercise session you will have physician consulting
	Once per 2 exercise sessions you will have physician consulting
	Once per 3 exercise sessions you will have physician consulting
	Once per 4 exercise sessions you will have physician consulting
Feedback and monitoring technology	Use of technology—feedback and monitoring of your exercises
	No technology—feedback and monitoring of your exercises
Program flexibility	Fixed exercise times
	Flexible exercise times
Health care premium reduction	No discount
	€50 discount
	€150 discount
	€450 discount

### Survey Format and Scenario Development

**Figure 1:**
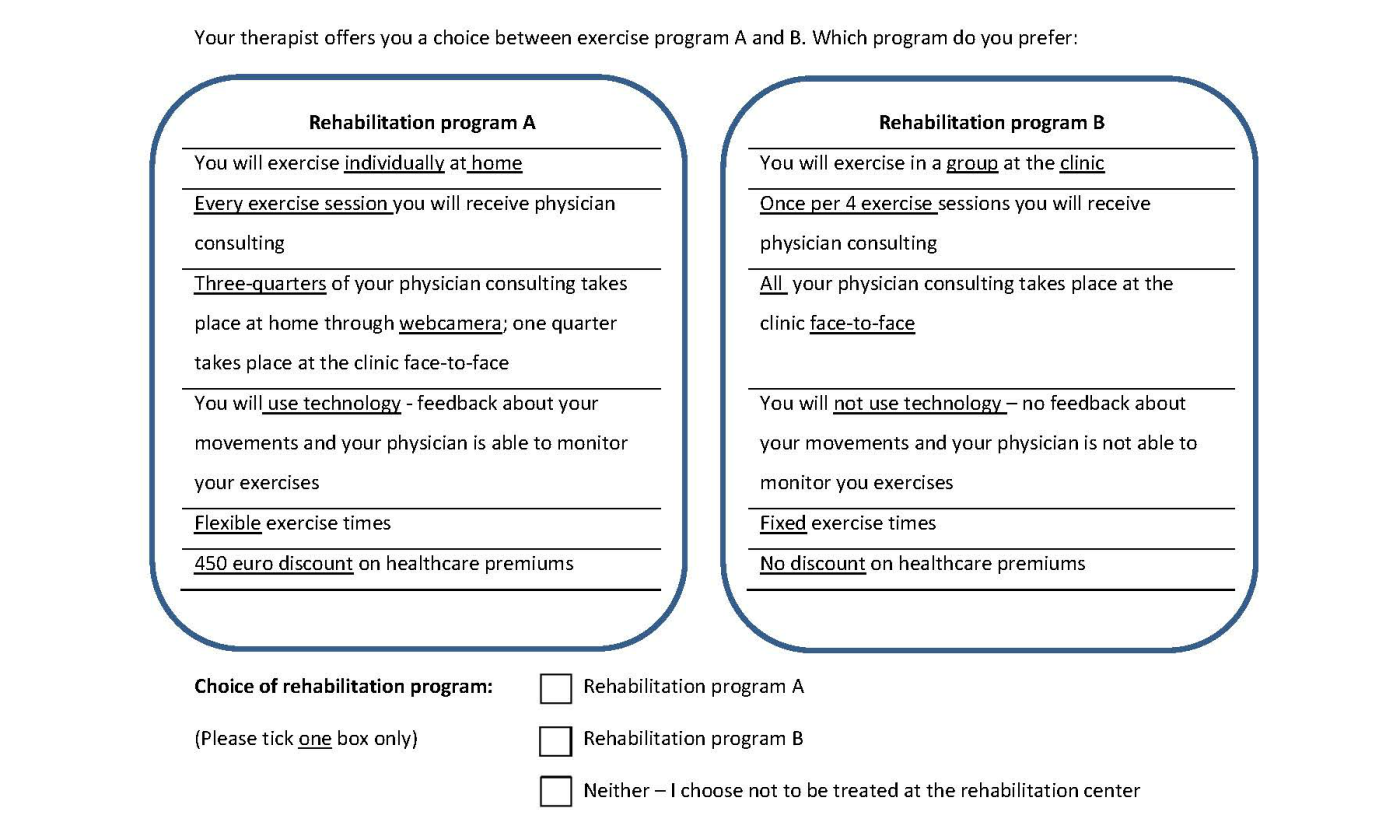
Questionnaire example.

Patients were offered 15 choice sets consisting of 2 telemedicine treatment scenarios and 1 opt-out scenario. They were asked to choose their preferred scenario. The scenarios comprised short statements based on the treatment attributes described earlier. Figure 1 represents a questionnaire example. The choice questions were designed to mimic the “real” choices, and as such, the opt-out option was included to ensure that the patients were not forced to make a choice between treatments when they might choose neither in practice. The attributes and levels in this study (4 attributes with 4 levels and 2 attributes with 2 levels) resulted in a total of 1024 hypothetical treatment scenarios. For practical reasons, not all of these could be presented to each respondent. Hence, we employed a commonly used D-optimal experimental design algorithm, which reduced the number of choice sets to the smallest number of choice sets required to generate statistically efficient preference estimates for the treatment attributes included. This resulted in a so-called fractional factorial design, using 3 versions of the questionnaire, which explored 45 choice sets in total. The resulting questionnaire design was orthogonal and balanced in terms of the number of times each level of an attribute was seen in a scenario. Subjects were randomly assigned to a questionnaire version. Sawtooth software (Sawtooth Software Inc) was used to design the choice tasks. Prior to choosing between treatment scenarios, all attribute levels were described to the patients.

### Survey Administration

Patients in this study were recruited from a waiting list of a rehabilitation center. These patients were waiting to enroll in a group-based supervised exercise program, which was part of the multidisciplinary pain rehabilitation program. In total, 300 questionnaires were administered per mail. Questionnaires were sent to patients’ home along with their invitation for a physician-led interview at the clinic. They were asked to return the completed questionnaire during the interview. Subjects were included if they were 18 years or older. Respondents did not receive incentives.

### Consistency Tests

In addition to the 15 choice sets, 3 fixed choice sets that were not included in analysis were presented to test patients’ response consistency and assess the internal validity of the stated-preference data. Validity was tested in 2 ways. The first was to include a choice set that presented a dominant scenario to assess whether patients chose the treatment scenario with the best treatment attributes. In this choice set, all treatment attributes of both scenarios were kept the same, except for WTA. Second, 2 choice sets were included that presented identical scenarios in reversed-order scenarios (“mirror set”). Patients who were inconsistent on both of these validity checks were excluded from the analysis.

### Model Estimation

The choice between the 2 alternative scenarios and the status quo can be seen as 2 choices simultaneously: first, the patient chooses between the status quo and telemedicine treatment, and second, the patient chooses between alternatives A and B. These 2 choices may depend on each other; that is, depending on the levels of the telemedicine treatment, the preference between status quo and telemedicine may change. We only observe the choice between the 2 telemedicine treatments when the status quo is not chosen; consequently, we will have complete observations of the first choice but a selected (censored) sample for the second choice. These types of data can be analyzed with a bivariate probit model with sample selection [40]. Patients’ utility for a telemedicine scenario is specified as linear in treatment attributes, and the utility of no treatment is an alternative- specific constant. Categorical test attributes were effects coded, and WTA was treated as a continuous variable. Accordingly, 2 functions were used (Textbox 1).

**Functions.**

V_treatment_=β_groupgym_×*D*_groupgym_+β_individualgym_×*D*_individualgym_+β_individualhome_×*D*_individualhome_+β_grouphome_×*D*_grouphome_+β_100%webcamera_ ×*D*_100%webcamera_+β_75%webcamera_×*D*_75%webcamera_+β_25%webcamera_×*D*_25%webcamera_+β_consultingeverysession_×*D*_consultingeverysession_+β_consultingper2sessions_ ×*D*_consultingper2sessions_+β_consultingper3sessions_×*D*_consultingper3sessions_+β_FeedbackMonitoringTechnology_×*D*_FeedbackMonitoringTechnology_+β_fixedsessions_ ×*D*_fixedsessions_+β_nodiscount_×*D*_nodiscount_+β_5%discount_×*D*_5%discount_+β_15%discount_×*D*_15%discount_+ε_treatment_

V_no-treatment_=(β_0_ + β_male_+β_<45 years_+β_education_+β_workhours_+β_internet_)×*D*_no-treatment_+ε_no-treatment_

The V_treatment_ β parameters represent relative importance weights, where larger values suggest more preferred attributes. Patient-specific characteristics are constant for any pair of treatment alternatives and cancel out the utility differences unless they are interacted with the uptake parameter. Therefore, patient characteristics were interacted with *D*_no-treatment_, which represents a dummy indicating that the respondents chose the “non-option.” The parameters indicate the effect of patients’ characteristics on telemedicine treatment uptake. The error terms ε_treatment_ and variable ε_no-treatment_ represent the part of the utility that is unobservable, and these error terms may be correlated with correlation ρ. The following patient characteristics were included in the final regression model: gender, age, education, Internet experience, and work hours.

The relative importance of the treatment attributes is represented by the coefficient estimates of the bivariate probit model. With these estimates, uptake of hypothetical telemedicine treatments can be predicted for different levels of incentives and other treatment attributes. For ease of presentation and interpretation, the model results were rescaled from 0 to 10 using a linear transformation of β coefficients from 0 (least desirable level) to 10 (most desirable level). Data were analyzed with heckprob function in Stata 11.2 (Statacorp).

### Scenario Comparison of Telerehabilitation Treatment

As well as the individual treatment attributes, patients’ preferences for 5 hypothetical telerehabilitation treatments were explored. These scenarios represented a continuum of health care settings ranging from clinic-based rehabilitation to home-based telerehabilitation with a focus on patient self-management and less physician involvement. All 5 scenarios were considered realistic treatment scenarios from a clinical perspective. One scenario represented conventional clinic-based rehabilitation. The conventional treatment consists of a supervised group-based exercise program at the rehabilitation clinic. The exercise program is part of a multidisciplinary pain rehabilitation program. In every session, exercises are supervised face-to-face by a rehabilitation physician. This conventional scenario was used to determine how patients valued the 5 telerehabilitation scenarios relative to conventional care. This was estimated with a willingness to accept value that represented a health care premium reduction in euros.

## Results

### Overview

We received 130 surveys that resulted in a total of 1950 observations from choice sets, with 13 observations missing. Patients who failed to pass both the validity checks were excluded from the analysis, which resulted in 104 usable questionnaires and a total of 1547 observations. The 104 respondents were spread fairly evenly across the 3 versions, with 42, 31, and 31 patients for versions 1, 2, and 3, respectively.

### Respondent Demographics

The majority of the research sample (mean age 43.8 years, SD 14.8) was female (66 out of 104) and had completed a middle-high education (51 out of 104 participants). The majority of the respondents were unemployed (69 out of 104 participants) at the time and had Internet access (97 out of 104 participants). Patients’ mean visual analogue scale (VAS) pain score was 6.3 and pain complaints varied in the lower back, hip, knee, joint, and neck areas and lasted longer than 6 months (Table 2).

### Relative Importance of the Treatment Attributes

The results of this study indicate that physician contact mode, feedback and monitoring technology, health care premium reduction, physician contact frequency, exercise location, and program flexibility are all significant determinants of patients’ treatment preference (*P*<.001). The sign and significance of the regression coefficients (Table 3) show that respondents preferred to have all physician counseling face to face. These face-to-face consultations were preferred over consultations that were offered either entirely or partly via remote video communication. Patients were relatively indifferent as to whether they had 25% or 75% of their consultation via video communication; however, having all consultations with video camera was the least preferred option. Furthermore, patients favored the use of feedback and monitoring technology while exercising and preferred to exercise at a gym location. In addition, they preferred physician contact every session and flexible exercise sessions and favored the highest discount on their health care premium. Conversely, respondents preferred not to undergo treatment that involved video consulting and minimized physician contact, exercising individually in the home environment without feedback and monitoring technology at fixed time frames. The attribute levels are generally well ordered, except for the attribute “consulting frequency.” Less frequent supervision (once per 4 exercise sessions) is preferred over more frequent supervision (once per 3 exercise sessions).

**Table 2:** Respondent characteristics.

Characteristics (N=104)		Mean (SD) or n (%)
**Gender, n (%)**		
	Female	66 (63.4)
**Age, years**		
	mean (SD)	43.8 (14.8)
	max, min	79, 20
**VAS pain score**		
	mean (SD)	6.3 (1.7)
	max, min	10, 2.1
**Education, n (%)**		
	Low	6 (5.8)
	Middle	50 (48.1)
	High	48 (46.2)
**Employment, n (%)**		
	Employed	35 (33.7)
**Internet, n (%)**		
	Yes	97 (93.3)

**Figure 2:**
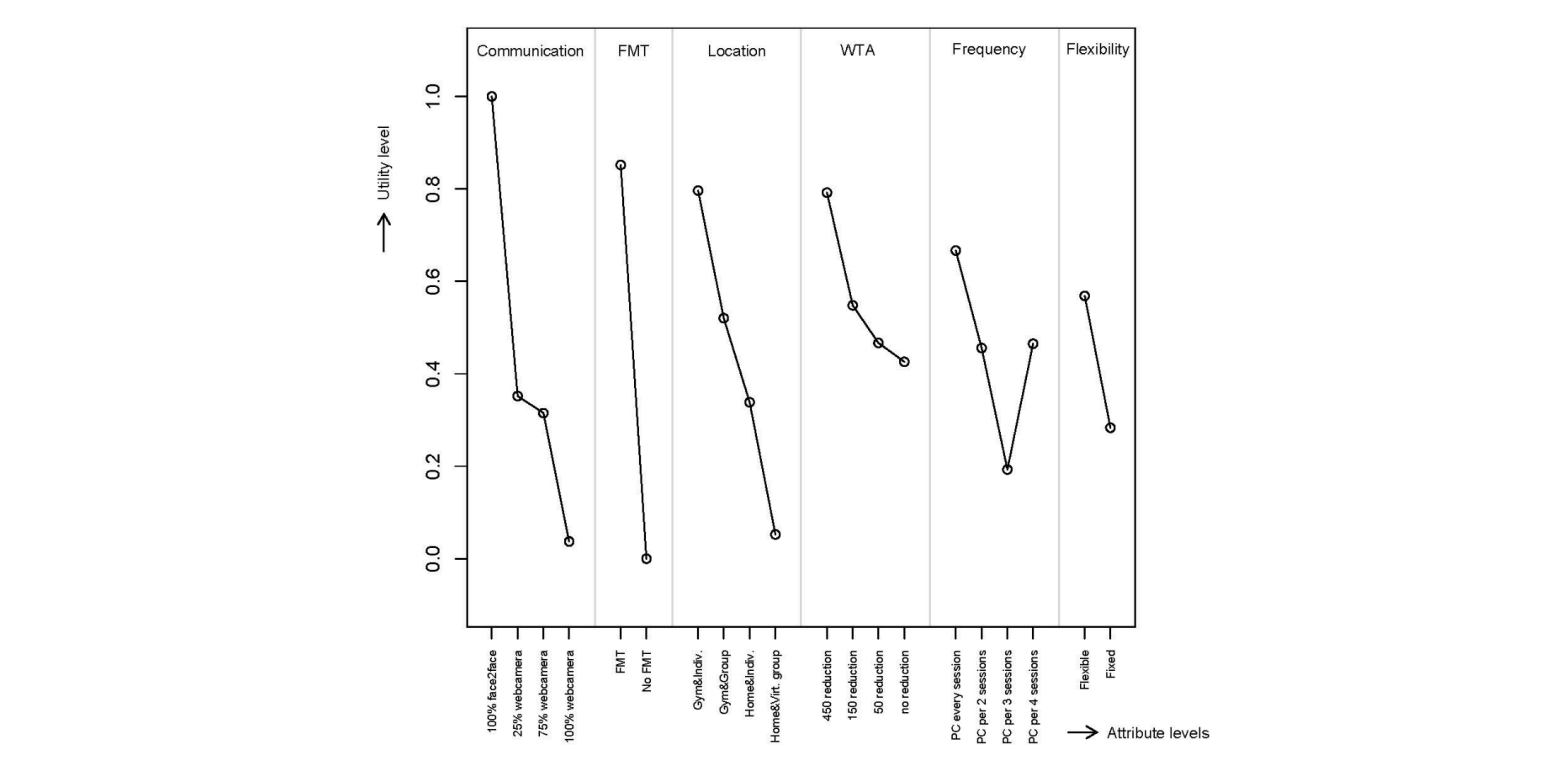
Relative importance of the attribute levels on a standardized scale.

Figure 2 illustrates the relative importance of the attribute levels on a standardized scale, with preference weights scaled between 0 and 1. For the most important attribute (physician contact mode), the most preferred level (100% face-to-face counseling sessions) is assigned a preference weight of 1. All other attribute levels are scaled relative to the most important attribute. Physician contact mode, the presence of feedback and monitoring technology, and exercise location were the most important attributes. The utility of moving from 100% face-to-face contact to 100% video consulting exceeded that for any other change between attribute levels. The smallest utility difference was between 25% video consulting versus 75% video consulting and €50 health care premium reduction and no health care premium reduction.

**Table 3:** Coefficient estimates of the bivariate probit model (N=1547).

Attribute level		Beta coefficient (standard error)	95% CI	*P* value
**Treatment mode and location**				
	Group at gym	.05 (0.05)	–0.04 to 0.14	.29
	Virtual group at home	–.20 (0.04)	–0.28 to –0.12	<.001
	Individually at gym	.20 (0.04)	0.11 to 0.28	<.001
	Individually at home	–.04 (0.05)	–0.14 to 0.05	.35
**Consulting frequency**				
	Every exercise session	.13 (0.04)	0.05 to 0.21	.001
	Once per 2 exercise sessions	.02 (0.04)	–0.06 to 0.09	.68
	Once per 3 exercise sessions	–.13 (0.04)	–0.20 to –0.05	.002
	Once per 4 exercise sessions	–.02 (0.04)	–0.10 to 0.06	.60
**Consulting mode**				
	100% Face-to-face consults	.31 (0.04)	0.22 to 0.39	<.001
	25% Video consults	–.04 (0.05)	–0.13 to 0.04	.32
	75% Video consults	–.06 (0.04)	–0.13 to 0.02	.17
	100% Video consults	–.21 (0.04)	–0.29 to –0.12	<.001
**Feedback and monitoring technology**				
	Yes	.22 (0.02)	0.19 to 0.26	<.001
	No	–.22 (0.02)	–0.26 to -0.19	<.001
**Flexibility exercise sessions**				
	Fixed	–.08 (0.02)	–0.12 to -0.03	<.001
	Flexible	.08 (0.02)	0.03 to 0.12	<.001
Health care premium reduction		.004 (0.001)	0.00 to 0.01	.001
**Decision of treatment (no treatment=0)**				
	Constant	1.59 (0.15)	1.30 to 1.87	<.001
	Gender	–.09 (0.10)	–0.29 to 0.12	.41
	Age >45 years	–.20 (0.10)	–0.40 to -0.01	.04
	Secondary education	.10 (0.13)	–0.16 to 0.36	.43
	Higher education	.13 (0.13)	–0.12 to 0.37	.31
	Internet	.21 (0.20)	–0.18 to 0.59	.29
	Work hours	–.34 (0.10)	–0.53 to -0.15	.001

### Comparison of Treatment Scenarios

Using the results of the bivariate probit model, the choice probabilities of 5 hypothetical telerehabilitation scenarios were explored (Table 4). These could be arrayed on a continuum from clinic-based rehabilitation to home-based telerehabilitation with a focus on patient self-management and less physician involvement, with scenario B as the most conventional scenario, E and F the least conventional, and C and D varying in between. Scenario A represented conventional clinic-based rehabilitation.

Table 4 shows that scenario C is preferred the most out of all treatment scenarios. This treatment scenario is considered an “intermediate” scenario that falls between conventional and telemedicine care. Patients are offered a clinical exercise environment with feedback and monitoring technology; however, face-to-face consulting with a physician is limited. Remarkably, scenario C is also the only scenario that outweighs the utility of conventional care (A). This demonstrates the willingness of patients to accept both a reduction in consulting frequency and face-to-face consulting when remote feedback and monitoring technology is offered.

Patients’ preferences for the 5 hypothetical telerehabilitation scenarios revealed that scenario F is the least preferred scenario. This scenario offers therapy at home with minimal physician supervision and requires a high level of patient self-management. Furthermore, the results demonstrated that conventional rehabilitation (A) is preferred over all home-based treatment scenarios varying in levels of monitoring and physician consulting (D-F). The model suggests that a reduction in health care premiums could raise the utility of these less preferred telerehabilitation treatments, which could increase future acceptance. For example, offering a reduction of 206.30 euros per year would make the least preferred scenario F equally attractive to conventional care. A smaller reduction (€70.70) is necessary to make scenario E equally attractive to conventional care.

**Table 4:** Utility of the different treatment scenarios (A-F; N=1547).

Treatment attributes	A	B	C	D	E	F
Location	Gym; group	Gym; group	Gym; individual	Home; individual	Home; virtual group	Home; virtual group
Communication	100% face-to-face	25% video	75% video	75% video	75% video	100 % video
Frequency	Every session	Every session	1×4 sessions	Every session	1×4 sessions	1×4 sessions
Feedback and monitoring technology	No	No	Yes	No	Yes	No
Flexibility	Fixed	Fixed	Fixed	Flexible	Flexible	Flexible
Health care premium reduction	None	None	None	None	None	None
Utility (SD) (Heckman)	0.18 (0.08)	–0.17 (0.08)	0.27 (0.08)	–0.42 (0.09)	–0.13 (0.08)	–0.73 (0.08)
WTA^a^ necessary to reach utility scenario A (euros)	–	79.3	0	136.6	70.7	206.3

^a^WTA: willingness to accept.

### Preferences for No Treatment

No treatment was preferred over treatment A or B in 136 observations, corresponding to 34 individuals who chose the “non-option.” Of these, 9 individuals did so on one occasion. One individual always chose the no treatment option. The parameter estimates for the patient characteristics age (*P*=.04) and work hours (*P*=.001) interacted with no treatment and were statistically significant. Older patients were more likely to choose the opt-out option than younger patients. Second, patients having a higher number of working hours were less likely to choose the opt-out option.

## Discussion

### Principal Findings

Although telemedicine is assumed to be improving efficient allocation of resources, its actual success depends on the patients’ acceptance and adherence. Therefore, future telemedicine services need to be designed with the patients’ perspective in mind. This study explored chronic pain patients’ preferences for telerehabilitation treatments using a discrete choice experiment and determined which future telerehabilitation design was preferred the most by chronic pain patients and which treatment attributes were most important to them. In addition, WTA was estimated to explore how patients valued telerehabilitation services relative to conventional rehabilitation and if they would be willing to trade health care premium discounts for more resource-efficient telerehabilitation treatments. Although DCEs are widely used in health care, this is the first study in the field of telerehabilitation estimating preferences for treatments to inform patient-centered treatment design.

Five hypothetical telerehabilitation scenarios were explored, which could be arrayed on a continuum from clinic-based rehabilitation to home-based telerehabilitation with a focus on patient self-management and minimal physician supervision. The most preferred treatment out of all 5 was an “intermediate” scenario that falls between conventional clinic-based rehabilitation and a telerehabilitation program with a focus on self-management and with no frequent face-to-face supervision. Patients preferred treatment outside the home environment, with a combination of video consultation and face-to-face consulting and the use of feedback and monitoring technology. Patients’ preference for an “intermediate” scenario demonstrates patients’ willingness to “trade” between treatment attributes and underscores the potential of the use of remote feedback and monitoring technology in chronic pain telerehabilitation. Patients were willing to accept less frequent physician consulting offered mainly through video communication, provided that they were offered assistance through remote feedback and monitoring technology and could exercise outside their home environment during flexible exercise hours. A key finding is that this “intermediate” scenario was preferred over conventional rehabilitation, which suggests that this scenario would make a feasible alternative to conventional care.

On the contrary, home-based telerehabilitation scenarios with minimal physician contact, provided entirely through video communication, and without the use of remote feedback and monitoring technology were preferred the least. This is an important finding, as a paradigm is emerging in which people with chronic disease are encouraged to take an active role in self-management and become actors in their own health care [41,42]. Offering remote feedback and monitoring technology as well as some physician face-to-face consulting would make home-based rehabilitation more attractive; however, it would not make these scenarios equally attractive to conventional rehabilitation. As such, to foster patient acceptance of home-based telerehabilitation with minimal physician supervision, other incentives are necessary to make these treatment scenarios more attractive.

WTA was estimated and demonstrated that chronic pain patients were willing to trade a reduction in health care premiums for less preferred treatment attributes, for example, less face-to-face physician consulting or a home-based treatment scenario. A reduction in health care premiums would make less preferred resource-efficient telerehabilitation scenarios with a focus on patient self-management equally attractive to conventional clinic-based rehabilitation. Ultimately, even a home-based telerehabilitation scenario with minimal physician consulting, the least preferred scenario out of all 5, could become an acceptable alternative to conventional clinic-based care if health care premium reduction is offered. However, these results must be interpreted with caution. Further research is necessary to explore whether, next to health care premium reductions, other strategies such as the use of motivational tools (eg, serious gaming) could increase the value of home-based telerehabilitation treatment.

In addition to the estimation of patients’ preferences for the various telerehabilitation scenarios, the importance of the individual treatment attributes was estimated. While all attributes impacted patients’ treatment preference, physician contact mode proved a key driver of preference for chronic pain rehabilitation with patients having a strong preference for some physician face-to-face contact. Treatment scenarios with partly remote physician video communication were preferred over scenarios that offered remote video communication only. The psychosocial nature of chronic pain treatment could be underlying this preference. In the treatment of chronic pain especially, the patient-physician communication plays an important role, as pain must be identified as a subjective phenomenon in the discussion [43] and both empathy and emotional support are considered essential [43,44]. Although touch is not necessary to convey empathy and establish a therapeutic bond [45,46] per se, a qualitative study in chronic pain patients established that some patients associated remote physician consultation with a loss of personal attention [47]. This same feeling of loss of personal attention was also found by Mair et al [48]. A physician’s inability to perform a hands-on physical examination during a remote consultation is also a cause for concern to some patients [46-49], which could also explain patients’ strong preference for physician face-to-face contact. Some patients consider face-to-face supervision an essential means to provide effective feedback and instruction. Furthermore, supervision during exercise may reduce patients’ insecurity and fear of exercising [50]. These findings indicate that integration of some face-to-face physician consultation is important to increase patient acceptance, which is consistent with other literature that found that attrition rates may be reduced by even minimal human contact [41]. A recent study of chronic pain patients suggests that Web-based chronic pain management intervention may be the most effective for patients with mild or moderate chronic pain who have better overall psychological and physical health. Individuals with numerous comorbidities, or spinal, neuropathic, or fibromyalgia pain, may require face-to-face contact, as this could be necessary in achieving optimal outcomes in pain management [51].

The importance that chronic pain patients place on feedback during exercise is also reflected in the value that patients place on the use of monitoring and feedback technology, which proved nearly as important as face-to-face physician contact. Strikingly, although none of the research sample had prior experience with the telemedicine technology, a factor that is associated with increased acceptance [52,53], the majority of our research sample preferred to use remote monitoring and feedback technology. Possibly, the use of the latest technology translates into “quality of care,” as some patients expect that the use of remote monitoring and feedback could provide even more accurate feedback than a therapist [47]. These results suggest that the lack of experience with the technology does not impede the acceptance of telerehabilitation and that, on the contrary, the use of innovative technology can be used as a way to increase acceptance of home telerehabilitation.

Treatment location proved a third important attribute, with patients having a preference for exercising individually outside the home environment. Patients attached great value to exercise in a clinic-based setting, either individually or in a group, rather than exercising in the home environment. Apparently, the hypothesized benefits that home treatment could bring to patients, for example, reduced transportation issues and easier translation of acquired skills, do not outweigh the disadvantages perceived by our study sample. Previous research with chronic pain patients demonstrated that a clinical environment can offer a more motivating environment for the patient and it creates an opportunity to get out of the house and meet other patients [47]. In addition, feelings of intrusion could be underlying the preference to exercise outside the home, since telerehabilitation brings clinical care into the “safe haven” of the home.

### Limitations

With regard to the reliability of the discrete choice experiment, some limitations of the study must be emphasized. First, the results might be limited in terms of the extent to which they could be generalized. Data were collected in a specific patient population, namely chronic pain patients waiting for their conventional rehabilitation to start. In addition, perceptions of patients who did not pass the consistency tests were disregarded. Little is known about how patients’ preferences regarding telemedicine change during treatment; therefore, we do not know whether patients’ possible insecurity at the start of their treatment had affected their telemedicine treatment preferences and whether this could explain why home-based telerehabilitation scenarios with a focus on self-management were preferred the least. Future studies should assess patients’ preferences at different points of time during rehabilitation, since preferences are likely to change over time and telerehabilitation treatments may need to be adjusted to the altering needs of patients during treatment. We also chose to include a non-option. This created a more realistic choice experiment, but also meant that we were limited in the exploration of the effect of patient demographics on patients’ preferences. Data revealed that both older patients and patients with a low education were more likely to choose the opt-out option. This could partly be attributed to the cognitive burden, for which discrete choice experiments have been criticized. In addition, we were not able to collect demographic information on nonresponders to determine whether there were systematic differences between responders and nonresponders. Future studies should further investigate the effect of patient demographics on treatment preference.

### Conclusions

A central aim of this study was to assess which treatment attributes were most important to chronic pain patients and to explore which telerehabilitation treatment was the most preferred. Physician contact mode, the use of feedback and monitoring technology, and exercise location were key drivers of patients’ treatment preferences. An “intermediate” treatment scenario consisting of attributes associated with both conventional rehabilitation and telerehabilitation was the most preferred. This demonstrated that patients were willing to accept less frequent physician consultation offered mainly through video communication, provided that they were offered feedback and monitoring technology and some face-to-face consultation and could exercise outside their home environment at flexible exercise hours. As such, telerehabilitation treatments that incorporate these attributes should be pursued as promising alternatives to conventional rehabilitation. Home-based telerehabilitation treatments with minimal physician supervision were the least preferred. However, offering health care premium reductions could make these treatments as attractive as conventional clinic-based rehabilitation.

## Abbreviations

DCE: discrete choice experiment
FMT: feedback and monitoring technology
WTA: willingness to accept
